# Collective prediction of protein functions from protein-protein interaction networks

**DOI:** 10.1186/1471-2105-15-S2-S9

**Published:** 2014-01-24

**Authors:** Qingyao Wu, Yunming Ye, Michael K Ng, Shen-Shyang Ho, Ruichao Shi

**Affiliations:** 1 Department of Computer Science, Shenzhen Graduate School, Harbin Institute of Technology, Shenzhen, China; 2 Shenzhen Key Laboratory of Internet Information Collaboration, Shenzhen, China; 3 Department of Mathematics, Hong Kong Baptist University, Hong Kong, China; 4 School of Computer Engineering, Nanyang Technological University, Singapore

**Keywords:** protein function prediction, protein-protein interaction network, collective classification

## Abstract

**Background:**

Automated assignment of functions to unknown proteins is one of the most important task in computational biology. The development of experimental methods for genome scale analysis of molecular interaction networks offers new ways to infer protein function from protein-protein interaction (PPI) network data. Existing techniques for *collective classification *(CC) usually increase accuracy for network data, wherein instances are interlinked with each other, using a large amount of labeled data for training. However, the labeled data are time-consuming and expensive to obtain. On the other hand, one can easily obtain large amount of unlabeled data. Thus, more sophisticated methods are needed to exploit the unlabeled data to increase prediction accuracy for protein function prediction.

**Results:**

In this paper, we propose an effective Markov chain based CC algorithm (ICAM) to tackle the label deficiency problem in CC for interrelated proteins from PPI networks. Our idea is to model the problem using two distinct Markov chain classifiers to make separate predictions with regard to attribute features from protein data and relational features from relational information. The ICAM learning algorithm combines the results of the two classifiers to compute the ranks of labels to indicate the importance of a set of labels to an instance, and uses an ICA framework to iteratively refine the learning models for improving performance of protein function prediction from PPI networks in the paucity of labeled data.

**Conclusion:**

Experimental results on the real-world Yeast protein-protein interaction datasets show that our proposed ICAM method is better than the other ICA-type methods given limited labeled training data. This approach can serve as a valuable tool for the study of protein function prediction from PPI networks.

## Background

We have witnessed a revolution in sequencing technologies in last decade. The biological sciences are undergoing an explosion in the amount of genome sequences. There are increasing interests about using computational methods to identify the biological functions of the protein sequences [1], as experimentally determining protein functions is time-consuming and it cannot catch up with the fast growth of newly found proteins [2].

Various studies have applied machine learning methods to protein data from biological experiments to predict the functions for unknown proteins. (e.g. [3,4]). Classical computational approaches for protein function prediction represent each protein as a set of features, and employ machine learning algorithms to automatically predict the protein function based on these features. The most well-established methods [5] are the BLAST [6] approach based on sequence, PROSITE [7] based on sequence motifs, and PFAM [8] based on profile methods.

In recent years, the development of experimental methods for genome scale analysis of molecular interaction networks offers new ways to infer protein function in the context of protein-protein interaction (PPI) network, wherein proteins and detected PPIs are represented by nodes and edges, respectively. The basic idea is that the direct interaction partners of a protein are likely to share similar biological functions [9]. Assignment of protein functions using PPI data has also been extensively studied, such as neighborhood counting based method [10], graph theoretic methods [11], hierarchical clustering-based methods [12] and graph clustering methods [13]. Although many efforts have been made in protein function prediction, most of them were based on either sequence similarity that ignores the protein interactions, or PPI information without using attributes derived from the content of protein sequence. The former method often fails to work if a query protein has no or very little sequence similarity to any proteins of known labels, the latter method has similar problem if there are insufficient relevant PPI information.

To explicitly use the information of the content of the data and the links information of the PPI network to improve the prediction performance, *collective classification *(CC) is proposed. It received considerable attentions in the last decade. Various CC algorithms has been proposed in the literature [14], such as the iterative classification algorithm (ICA) [15], Gibbs sampling (Gibbs) [16], and variants of the weighted-vote relational neighbor algorithm (wvRN) [17]. Here, we focus on ICA-type approaches, which consist of a local classifier, such as *k*NN, to infer the class labels of related instances. The key idea is to construct new relational feature vectors by summarizing the label information from neighborhood nodes, and then use the relational features together with the attribute features derived from the content of data to learn local classifiers for prediction.

**Figure 1:**
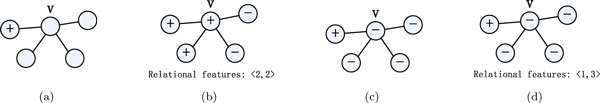
**An example of ICA algorithm learning with limited labeled data**. (a) initial state, train classifier *M_A _*and classify *V^U^*; (b) Compute relational features *X_R _*, train classifier *M_AR_*; (c) re-predict *V^U ^*(use *M_AR_*); (d) re-compute relational features *X_R _*. ICA repeats step c and step d until a fixed iteration number.

Figure 1 is an illustration of how ICA proceeds. In Figure 1(a), an attribute-only classifier *M_A _*induced from using only the attribute features is first learned to estimate the classes of unlabeled instances. The algorithm then employs an aggregation function to compute the relational features by counting the number of neighbors with respect to each label. Once the features are constructed, a collective classifier, *M_AR_*, is learned using both the attribute features and relational features (Figure 1(b)); The algorithm repeats step c and step d to make new prediction for unlabeled instances (Figure 1(c)), and to update the relational features based on the new generated predictions (Figure 1(d)). The ICA-type of algorithms usually assume a separate training graph with abundant labeled data. However, in many applications such as protein function prediction problems, the number of labeled protein data is actually very limited and very expensive to obtain. In this situation, most data have no connection to labeled data, and supervision knowledge cannot be obtained from the local connections (as illustrated in Figure 1(a)). As a result, the collective classifier *M_AR _*learned from these networks may suffer a reduction in the classification performance.

This paper describes an effective Markov chain based CC algorithm (ICAM) to tackle the label deficiency problem in CC for protein function prediction from PPI networks. Our idea is to model the classifier *M_AR _*via the Markov chain with restart. The Markov chain model computes the ranks of labels to indicate the importance of a set of labels to an instance by propagating the label information in a graph constructed from labeled and unlabeled data. The ICAM algorithm further refines the Markov chain model using an ICA framework to generate the possible labels for a given instance. By these techniques, *M_AR _*can be learned more effectively. Experiments on the realworld Yeast PPI datasets have demonstrated that our proposed ICAM method improves the classification performance when compared with the ICA-type CC methods. The main contributions of this paper are as follows.

• We study the label deficiency problem of collective classification (CC) and show that the protein function prediction problem from PPI networks can be formulated as a CC task.

• We extend the ICA-type CC algorithm and propose the ICAM algorithm to leverage the unlabeled portion of the data to improve the classification performance of CC via the Markov chain with restart.

• We demonstrate the effectiveness of our proposed ICAM algorithm using the Yeast benchmark datasets. We find that ICAM leads to significant accuracy gains compared to other ICA-type methods when there are limited numbers of labeled data available.

## Methods

### Preliminaries

Assume that the PPI network data are represented as a graph *G*(*V*, *E*, *X_A_*, *Y*, *c*), where *V *is a set of nodes, *E *is a set of edges representing the interactions between the instances. Each instance/node *v_i _*∈ *V *is described by an attribute vector *x_i _*∈ *X_A_*. Each *Y_i _*∈ *Y *is a set of labels for *v_i_*, and *c *is the number of possible labels. Assume that we have a set of labeled nodes *V^K ^*⊂ *V *with known labels *Y^K ^*= {*Y_i_|v_i _*∈ *V^K^*}, and the task is to predict the labels *Y^U ^*for unlabeled nodes *V^U ^*= *V - V^K^*. In this paper, we are primarily interested in generating a ranking of possible labels for a given protein such that its correct functions receive higher ranking than the less unlikely one.

### The ICAM algorithm

Inspired by the ICA approach, we introduce the ICAM algorithm for collective classification. The algorithm is summarized in Algorithm 1. Similar to the ICA framework, the ICAM algorithm has two parts as follows: *bootstrap *and *iterative inference*. The *bootstrap *part learns an attribute-only classifier *M_A _*from the known nodes, and uses *M_A _*to predict labels for the unknown nodes *V^U ^*(step 1-2). In the iterative inference part, the relational features *X_R _*are updated based on the estimated class labels of data (step 4). Specifically, *X_R _*of the (*i *+ 1)-th iteration is based on the known and predicted labels from the *i*-th iteration. Next, the algorithm trains a collective classifier *M_AR _*using both attribute features *X_A _*and relational features *X_R _*to compute the labels for unlabeled data. The iterative process stops when the predictions of *M_AR _*are stabilized or a fixed number of iteration is reached.

An important component of the ICA algorithm is to build the relational features that summarizes the relational information, and to construct new feature vectors to train the classifier *M_AR_*. For instance, Neville et al. [15] summarize the labels of neighboring nodes as relational features as illustrated in Figure 1(b), where node "B" has two positive neighboring nodes and two negative neighboring nodes. Here, the relational features is "¡2, 2¿", and then "¡2, 2¿" is appended onto the original feature vector, <*x*_i,1_, *x*_i,2_, ⋯>, as new features, " <*x*_i,1_, *x*_*i*,2_, *⋯*, 2, 2 >". ICA-type CC methods usually increase accuracy for network data using a large amount of labeled data to train *M_AR_*. In this scenario, the supervision knowledge can be effectively propagated in the network and improve the learning accuracy [18]. However, the labeled data are time-consuming to obtain and the number of labeled data is very limited. Most of the nodes may not link to the labeled nodes, as illustrated in Figure 1(a). As a result, the prediction accuracy of the collective classifier *M_AR _*will be decreased greatly.

**Algorithm 1 ***ICAM *(*V*, *E*, *X_A_*, *Y^K^*, *n*)

**Input**:

*V *= nodes, *E *= edges, *X_A _*= attribute feature vectors, *Y^K ^*= labels of known nodes, *n *= # of iterations,

**Procedure**:

1: *M_A _*= *learnClassifer*(*X_A_*, *Y^K^*);

2: $Y^{U}=predict\left(M_{A},X_{A}^{U}\right);$

3: **for ***t *= 0 to *n ***do**

4:    *X_R _*= *aggregation*(*V*, *E*, *Y^K ^*∪ *Y^U^*);

5:    Re-train *M_AR _*= *learnClassifer*(*X_A_*, *X_R _*, *Y^K^*);

6:    $Y^{U}=predict\left(M_{AR},V,E,X_{A}^{U},X_{R}^{U},Y^{K}\right);$

7: **end for**

8: **return ***Y^U^*

In our ICAM algorithm, we assume that the attribute features *x_i _*and the relational features *r_i _*are conditionally independent given the class label *Y*_*i *_[19]. We then use two distinct classifiers to make two separate predictions for attribute features *X_A _*and relational features *X_R_*. The prediction is given as follows:

$$\begin{array}{ll} p\left(Y_{i}|x_{i},r_{i}\right) & =\frac{p\left(x_{i}|Y_{i}\right)p\left(r_{i}|Y_{i}\right)p\left(Y_{i}\right)}{p\left(x_{i},r_{i}\right)}\;\\ & =\frac{\frac{p\left(Y_{i}|x_{i}\right)p\left(x_{i}\right)}{p\left(Y_{i}\right)}\frac{p\left(Y_{i}|r_{i}\right)p\left(r_{i}\right)}{p\left(Y_{i}\right)}p\left(Y_{i}\right)}{p\left(x_{i},r_{i}\right)}\;\\ & =\gamma \frac{p\left(Y_{i}|x_{i}\right)p\left(Y_{i}|r_{i}\right)}{p\left(Y_{i}\right)}\;\\ & \; \end{array}$$

where *γ *is a constant independent of *Y_i_*. The attribute classifier to estimate *p*(*Y_i_|x_i_*) is referred to as *M_A_*, and the relational classifier to estimate *p*(*Y_i_|r_i_*) is referred to as *M_R_*.

There are two main advantages of this prediction method. First, this method allows us to train classifiers *M_A _*and *M_R _*for attribute features *X_A _*and relational features *X_R _*in parallel. Second, in the collective inference process, the classifier *M_R _*can be re-trained in each iteration based on the re-constructed relational features *X_R _*to improve the prediction accuracy of the collective classifier *M_AR_*.

Various traditional supervised learning methods can be used to train *M_A _*and *M_R _*where the classifier, such as *k*NN, naive bayes and logistic regression [16,20], is learned from a separate training data with a large amount of labeled data. However, when dealing with label deficiency problem in PPI networks, we propose to use transductive learning method for acquiring additional information from unlabeled data to improve the classification performance. Specifically, we set up Markov chain based learning models to estimate *p*(*Y_i_|x_i_*) and *p*(*Y_i_|r_i_*).

### Markov chain based learning

The Markov chain based learning model is based on the idea of random walk with restart. We note that there are many learning tasks using random walk techniques such as protein network cluster discovery [21], community discovery [22], multi-instance multi-label learning [23], and transfer learning [24]. The idea of random walk with restart is to consider a random walker that starts from labeled nodes, and iteratively transmits to its neighborhood with probabilities proportional to their edge weights. At each step, it has a probability *α*(0 *< α <*1) to return back to labeled node. The steady-state probability that the walker will finally stay at node *j *is the relevance score of node *j *with respect to the labeled nodes [25]:

u=(1-α)Pu+αq

where **u **= [*u_j_*] is the steady-state probability of relevance scores of different nodes, **P **is the affinity matrix associated with the instances in Markov chain transition probability graph, and **q **is the label distribution vector containing the elements of labeled instances being 1 and 0 for others. Here, the steady-state probability (relevance score of the instances) captures the global structure of the graph and relationship between the nodes. The advantage of this random walk procedure is that it converges to a unique solution for any initial **u**(0). The process converges fast, needing just a few iterations. The random walk and related methods have been shown to have good performance on the learning tasks mentioned above. In the following, we introduce the learning of *M_A _*via the Markov chain with restart using all the instances (both labeled and unlabeled). The process of learning *M_R _*is similar.

Given the constructed attribute feature vector *x_i _*∈ *X_A _*for a node *v_i _*∈ *V*, pairwise affinity $A\in {\mathbb{R}}^{m\times m}$ between the nodes based on relational information are computed using the Gaussian kernel function as follows

(1) $$a_{i,j}=\text{exp}\left[\frac{-||x_{i}-x_{j}|{|}_{2}}{2\sigma^{2}}\right],$$

where *||x_i _- x_j_|| *is the Euclidean distance between the *i*-th feature vector and the *j*-th feature vector in *X_A_*. The parameter *σ *is a positive number to control the linkage in the manifold [26]. The *m*-by-*m *matrix **A**, with its (*i*, *j*)-th entry given by *a*_*i*,*j*_, is always nonnegative. Similar to (1), using the Gaussian kernel to *r_i _*∈ *X_R _*leads to the affinity matrix **R **for relational features. We then set up Markov chain models for classifiers *M_A _*and *M_R _*based on **A **and **R**, respectively.

For the classifier *M_A_*, we construct an *m*-by-*m *Markov transition probability matrix **P **by normalizing the entries of **A **with respect to each column, i.e., each column sum of **P **is equal to one, $\sum_{i}^{}\left[P\right]{\;}_{i,j}=1$. For such **P**, we model the probabilities of visiting the other instances from the current instance in a Markov chain transition probability graph. We construct a transition probability graph, all the labeled and unlabeled instances are linked together. Intuitively, a random walker starts from nodes with known label to propagate labels among labeled instances to the other unlabeled instances. The walker iteratively visits its neighborhood of nodes with the transition probability graph based on **A**.

Next we use the idea in topic-sensitive PageRank [27] as a Markov chain with restart [25] to solve the learning problem. The random walker has a probability of *α *to return to labeled instances at each step. It can be interpreted that during each iteration each instance receives the label information from its neighbors via the random walk, and also retains its initial label information. The parameter *α*specifies the relative amount of the information from its neighbors and its initial label information. Using this approach, we compute the steady state probabilities that the random walker finally stay at different instances. These steady state probabilities give ranking of labels to indicate the importance of a set of labels to an unlabeled instance.

More formally, we adopt the following equation:

(2) U=(1-α)PU+αQ,

to compute the steady probabilities **U **= [**u_1_**, **u_2_**, ⋯, **u_c_**] (*m*-by-*c *matrix) according to **P **and **Q **= [**q_1_**, **q_2_**, ⋯ , **q_c_**] (*m*-by-*c *matrix) which is the assigned probability distribution vector of the class labels that are constructed from the labeled data. The restart parameter 0 *< α <*1 controls the importance of the assigned probability distributions in the labeled data to the resulting label ranking scores of instances. Given the training data, one simple way to construct q*_d _*is using a uniform distribution on the instances with the label class *d*. The summations of the entries of q*_d _*is equal to 1. More precisely,

(3) $${\left[qd\right]}_{i}=\left\{\begin{array}{cc} 1/l_{d}, & \text{if}\;d\in Y_{i}\\ 0, & \text{otherwise}\text{.} \end{array}\right)$$

where *l_d _*is the number of instances with the label class *d *in the training data.

The steady probability distribution vector **U **is solved by the iteration method with an initial matrix **U**_0 _where each column is a probability distribution vector. The overall algorithm is summarized in Algorithm 2.

**Algorithm 2 **Markov Chain based Classifer

**Input**: **P**, **Q **and **U**_0_, α, and the tolerance ϵ

**Output**:the steady probability distribution matrix **U**

**Procedure**:

1: Set *t *= 1;

2: Compute **U***_t _*= (1 - *α*)**PU**_*t*-1 _+ *α*Q;

3: If ||**U***_t _*- **U**_*t*-1_|| < ϵ, then stop, set **U **= **U***_t_*; otherwise set *t *= *t *+ 1 and goto Step 2.

## Experimental results

In this section, we compare the performance of ICAM algorithm with other ICA-type collective classification algorithms: ICA, Gibbs and ICML. We show that the proposed algorithm outperforms these algorithms given limited number of labeled training data.

### KDD Cup 2001 data and baselines

The first experiment is conducted for Yeast gene function prediction from KDD Cup 2001 [28]. The dataset includes 1,243 genes and 1,806 interactions among the pair of genes encoding the proteins physically interact with one another. These interaction relationships are symmetric. The protein functions are autocorrelated in this dataset and a subset of the data have been withheld for testing. The task is to predict the functions of the proteins encoded by the genes. There are 14 functions and a protein can have one (or several) function(s).

We compare our proposed method with the following three baseline learners:

1. **ICA**. The Iterative Classification Algorithm (ICA) algorithm proposed by Neville et al. [15] is one of the simplest and most popular CC methods that is frequently used as baseline for CC evaluation in previous studies. For multi-label problem, we transform it into multiple single-label prediction problems using one-against-all strategy and employ ICA to make prediction for each single-label problem.

2. **Gibbs**. This baseline is another ICA-type CC algorithm using the ICA iterative classification framework. In each iteration, Gibbs re-samples the label of each node based on the estimated label distribution [16]. We also use one against-all strategy to convert the multi-label problem into multiple single-label problems for the Gibbs algorithm.

3. **ICML**. This baseline is a multi-label CC algorithm proposed by Kongetal. [29]. ICML extends the ICA algorithm to multi-label problems by considering dependencies among the label set in the iteration process.

In the experiments, we use *k*NN as node classifier for ICA, Gibbs and ICML. The parameter *k *was automatically selected in the range of 10 to 30 at an increment of 5 using 3-fold cross validation on the training set. For the proposed ICAM method, we learn the classifiers *M_A _*and *M*_*R *_using Markov chain based models to perform separate predictions. We set the value of *α *in the Markov chain model to be 0.95 as suggested in [23].

### Evaluation criteria

We evaluate the performance of our proposed method with four multi-label evaluation measures: *average precision*, *coverage*, *ranking loss*, and *one-error*. They are commonly used for multi-label learning algorithm evaluation.

Given a multi-label dataset *D *= {(*x_i_*, *Y_i_*)*|*1 *≤ i ≤ m*}, where $x_{i}\in X$ is an instance and $Y_{i}\subseteq Y$ is the true labels of *x_i_*, and *Y_i _*= (*Y*_*i*1_, *Y*_*i*2_, ..., *Y*_*ic*_) ∈ {0, 1}*^c^*. Here *x_i _*belongs to the *j*-th label when *Y_ij _*= 1, otherwise *Y_ij _*= 0, and *c *is the number of possible labels. The evaluation measures are defined using the following two outputs provided by the learning algorithms: *s*(*x_i_*, *l*) returns a real-value that indicates the confidence for the class label *l *to be a proper label of *x_i_*; *rank_s _*(*x_i_*, *l*) returns the ranks of class label *l *derived from *s*(*x_i_*, *l*).

*Coverage *[30] evaluates how far we need, on the average, to go down on the list of labels in order to cover all the true labels of an instance:

$$\text{coverage(}f\text{) = }\frac{1}{m}\overset{m}{\underset{i=1}{\sum }}\underset{l\in Y_{i}}{\text{max}}\;rank_{s}\left(x_{i},l\right)-1.$$

*Ranking loss *[30] evaluates the average fraction of label pairs that are reverse ordered for the instance:

$$rloss\text{(}f\text{) = }\frac{1}{m}\overset{m}{\underset{i=1}{\sum }}\frac{1}{\left|Y_{i}||{Ȳ}_{i}\right|}.|R_{i}|,$$

where $R_{i}=\left\{\left(l_{\text{1}},l_{\text{2}}\right)|h\left(x_{i},l_{\text{1}}\right)\leq h\left(x_{i},l_{\text{2}}\right),\left(l_{\text{1}},l_{\text{2}}\right)\in Y_{i}\times {Ȳ}_{i}|\right\}$.

*One-error *[30] evaluates how many times the top-ranked label is *not *in the set of true labels of the instance. Define a classifier *H *that assigns a single label to an instance *x_i _*by $H\left(x_{i}\right)=\text{arg}{\text{max}}_{l\in Y}h\left(x_{i},l\right)$, then the one-error is

$$one-error\left(H\right)=\frac{1}{m}\overset{m}{\underset{i=1}{\sum }}\left[\left[H\left(x_{i}\right)\notin Y_{i}\right]\right].$$

*Average precision *[30] evaluates the average fraction of labels ranked above a particular label *l *∈ *Y *in *Y*:

$$avgprec\left(f\right)=\frac{1}{m}\overset{m}{\underset{i=1}{\sum }}\frac{1}{\left|Y_{i}\right|}\underset{y\in Y_{i}}{\sum }\frac{\left|P_{i}\right|}{rank_{s}\left(x_{i},l\right)},$$

where $P_{i}=\left\{l^{\prime }\in Y_{i}|rank_{s}\left(x_{i},l^{\prime }\right)\leq rank_{s}\left(x_{i},l\right)\right\}$.

The smaller the value of *coverage ranking loss *and *one-error*, the better the performance. As for *average precision*, the bigger the value the better the performance, we report the results of 1-*average precision*. Thus, for all evaluation metrics, the smaller the value the better the performance.

### Results on KDD Cup 2001 data

In this experiment, we test the performance of our proposed ICAM algorithm on the KDD Cup 2001 dataset. We randomly select 50% of data as training set, and use the remaining 50% of data as test set. The experiment is conducted 10 times by randomly selected training/test split (each with a different random seed), and we report the results of mean as well as standard deviation of each compared algorithm. The mean as well as standard deviation of each compared method over the same 10 trails are reported.

Table 1 shows the performance of each compared method on the Yeast protein dataset. The best performance achieved among different compared algorithms is marked in bolded. The results show the competitiveness of the ICAM method against other learning methods. Difference evaluation measures for the learning performance are used in the experiments. One algorithm rarely outperforms another algorithm in all criteria. For example, a method that is optimal for instance *ranking loss *usually does not perform well in *coverage *or *one-error *[31]. In the experiments, we find that ICAM is able to produce better results across all evaluation metrics. These results are impressive and imply that the ICAM algorithm is a good collective classification method for protein function prediction.

**Table 1:** The performance (mean ± standard deviation) of compared algorithms on the Yeast protein dataset.

Methods	Coverage	Ranking Loss	One-error	1-Average Precision
ICA	4.217 ± 0.273	0.140 ± 0.013	0.042 ± 0.005	0.155 ± 0.005
Gibbs	4.319 ± 0.195	0.148 ± 0.008	0.043 ± 0.005	0.154 ± 0.006
ICML	4.409 ± 0.091	0.153 ± 0.006	0.043 ± 0.007	0.162 ± 0.006
ICAM	**3.748 **± **0.164**	**0.100 **± **0.008**	**0.041 **± **0.005**	**0.151 **± **0.005**

**Figure 2:**
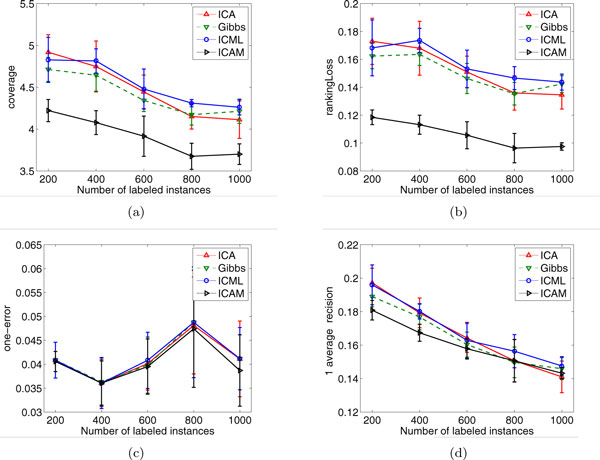
**The performance of different algorithms on the Yeast protein dataset with varying number of labeled instances**.

We also test the performance of different comparable algorithms with different number of labeled instances ranging from 200 to 1000 with an increment of 200. For example, we randomly pick up 200 instances as training data. The remaining data is used for testing. The experiment is conducted 10 times by randomly selecting training/test split. We report the results of mean as well as standard deviation of each compared algorithms. Figure 2 shows the performance of ICAM and other learning methods with respect to different number of labeled instances.

We can see from the figure that ICAM (the black line) has the best performance in general. ICAM outperforms other algorithms using different number of training data, especially when the size of training data is small. Specifically, ICAM achieves *coverage *improvement of 0.4916 over the second best method Gibbs (ICAM:4.2213 versus Gibbs:4.7129) and achieves 0.0439 improvement on *ranking loss *(ICAM:0.1184 versus ICML:0.1623) when the number of training instance is 200. As the size of training data increases, ICAM consistently achieves better performance than other learning algorithms across all evaluation criteria.

We find that ICAM outperforms the other ICA-type methods substantially in terms of *coverage*. On the other hand, ICAM only slightly outperforms other methods in terms of *one-error*. We note that *one-error *and *coverage *are two different quantitative measures. One-error evaluates how many times the *top*-ranked label is not in the set of possible labels. Thus, if the goal of a prediction system is to assign a single function to a protein (single-label classification), the one-error is identical to test error. Whilst coverage measures how far we need, on the average, to go down on the list of the labels in order to cover all the possible labels assigned to a protein. Coverage is loosely related to precision at the level of perfect recall [30]. The experimental results indicate that the top-rank label predictions from other ICA-type methods are as accurate as those from ICAM, but the predictions from ICAM are more complete than other ICA-type methods. A reasonable explanation for this finding is that the ICA-type methods focused on the single-label setting. In this case, the multi-label problem is first transformed into multiple single-label prediction problems, and then the ICA-type methods use independent classifiers induced from labeled training data for each problem. Nevertheless, ICA-type approaches ignore the effect of unlabeled data and the interdependence of the protein functions. On the other hand, our proposed ICAM approach is based on Markov chain based transductive learning method that uses both label and unlabeled data for label propagation. The Markov chain based method takes the correlation of the classes into account to effectively compute ranking of labels to an instance. Therefore, ICAM provides an opportunity to leverage the individual ICA-type classifiers to achieve higher coverage of predictions.

### Results on KDD Cup 2002 data

To validate the effectiveness of the proposed method when there are only a limited number of positive labeled training data, we conduct additional experiments on a relatively large scale Yeast dataset from KDD Cup 2002. It consists of 4507 instances (i.e., genes) from experiments with a set of cerevisiae (Yeast) strains. Each instance is described by various types of information that characterize the gene associated with the instance. The data sources for describing the instances include abstracts from the scientific literature (MEDLINE), gene localization and functions. We represent each instance by a feature vector with 20545 dimensions. The pairs of genes whose encoded proteins physically interact with one another. Such protein-protein interaction network consists of 1218 links.

Each instance is labeled with one of three class labels "nc", "control" and "change". The "change" label indicates instances in which the activity of the hidden system was significantly changed, but the activity of the control system was not significantly changed. The goal of the KDD Cup 2002 task is to learn a model that can accurately predict the genes that affect the hidden system but not the control system. In this case, the positive class consists of those genes with "change" labels and the negative class consists of those genes with either "nc" or the "control" label. This partition is highly imbalanced. The rate of positive instances is only 1.2%. Therefore, we base our evaluation analysis on Receiver Operating Characteristic (ROC) curves, which reflect the true positive rate of a classifier as a function of its false positive rate. ROC curves are commonly used for evaluating highly skewed binary classification problems. Recent study has shown that ROC curves have a deep connection to the precision-recall (PR) curves [32].

**Figure 3:**
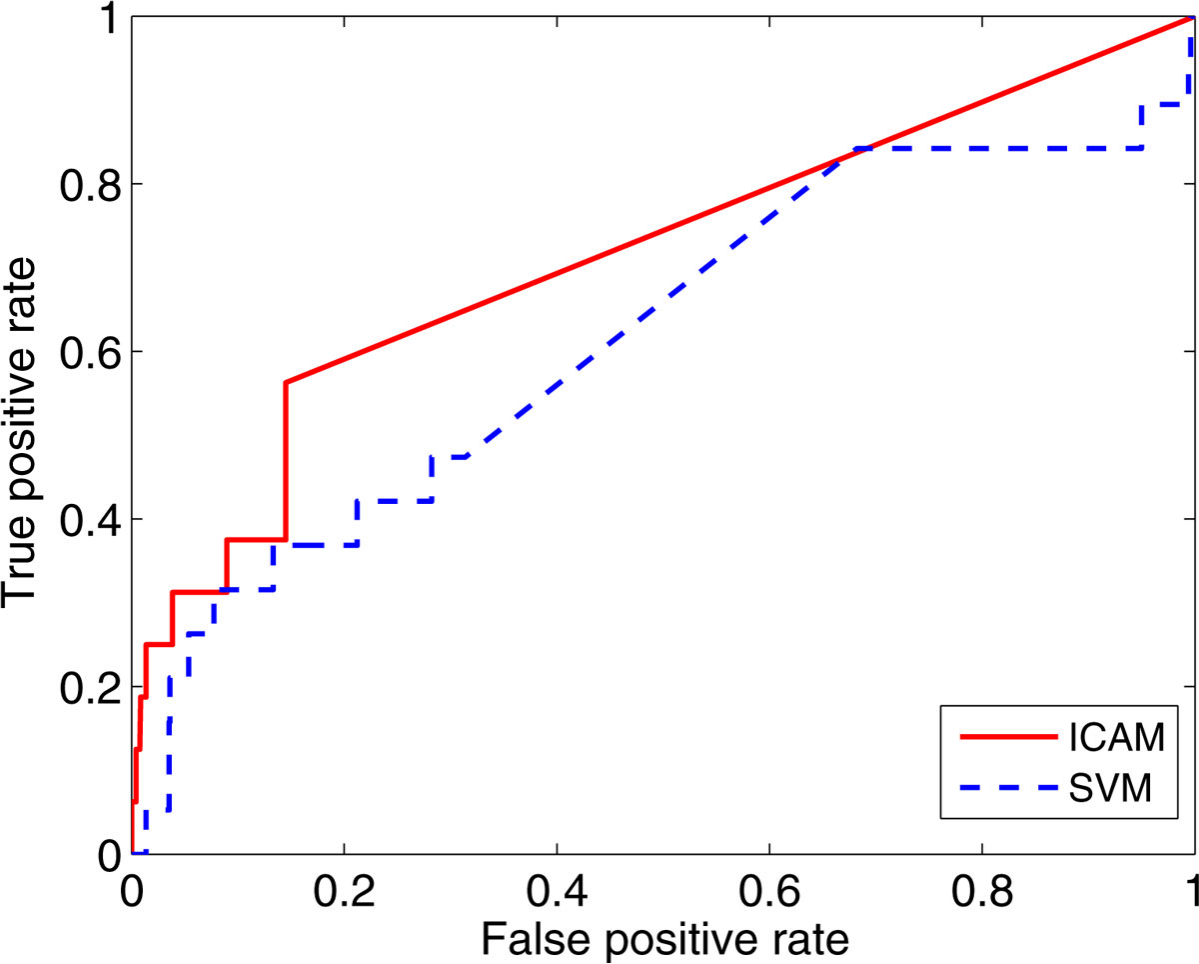
**ROC curve of baseline SVM and our ICAM method**.

To evaluate the performance of our ICAM algorithm, we compare it with the linear kernel SVM method that implemented by LIBSVM [33]. Figure 3 shows the results of ROC curves on the KDD Cup 2002 task for ICAM and SVM. The *x*-axis and *y*-axis of the figure refer to the false positive rate and true positive rate respectively. We see from the figure that our ICAM (the red curve), outperforms the SVM method (the blue curve) in general. ICAM achieves improvement of 10.0% (0.713 versus 0.613) on area under the ROC curves. The experimental results imply that the proposed ICAM method is able to deliver better performance in the situation of small positive labeled data size.

## Experiments on collaboration networks

In this section, we compare the performance of the proposed ICAM algorithm with other collective classification algorithms on 2 multi-label collaboration networks datasets to validate the effectiveness of the proposed method more thoroughly. These collaboration networks datasets are collected from the DBLP computer science bibliography website, and used in prior work to study the multi-label collective classification problems [29]. Their characteristics are listed in Table 2. Specifically, we extract DBLP coauthorship networks that contain authors who have published papers during the years 2000-2010 as the nodes of the networks, and link any two authors who have collaborated with each other. At each node, we extract a bag-of-words representation of all the paper titles published by the author, and used it as the attributes of the node. Each author has one (or multiple) research topic(s) of interests from 6 research areas. The representative conferences from each area are selected as class labels. If an author has published papers in any of these conferences, we assume the author is interested in the corresponding research class. The task is to classify each author with a set of multiple research classes of interest. The conferences corresponding to the class labels of two datasets (DBLP-A and DBLP-B) are given as follows.

The classes of DBLP-A are as follows:

1 Database: ICDE, VLDB, SIGMOD, PODS, EDBT

2 Data Mining: KDD, ICDM, SDM, PKDD, PAKDD

3 Artificial Intelligence: IJCAI, AAAI

4 Information Retrieval: SIGIR, ECIR

5 Computer Vision: CVPR

6 Machine Learning: ICML, ECML

The classes of DBLP-B are as follows:

1 Algorithms & Theory: STOC, FOCS, SODA, COLT

2 Natural Language Processing: ACL, ANLP, COLING

3 Bioinformatics: ISMB, RECOMB

4 Networking: SIGCOMM, MOBICOM, INFOCOM

5 Operating Systems: SOSP, OSDI

6 Parallel Computing: POD, ICS

**Figure 4:**
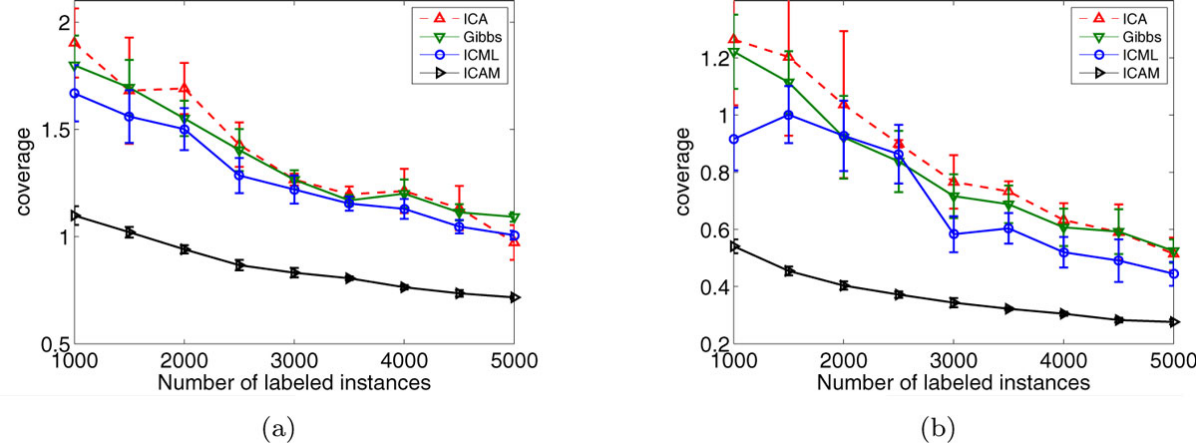
**The coverage performance of different algorithms with varying number of labeled instances**: (a) DBLP-A dataset; (b) DBLP-B dataset.

We test ICAM and other ICA-type algorithms with different number of labeled instances from 1000 to 5000 with an increment of 500. The average results as well as standard deviation of a 10-time data split are given in Figure 4. The experimental results are in concordant with our previous study. We observe that ICAM consistently outperforms the other ICA-type methods on these two datasets, especially when there are only limited number of labeled instances, i.e. larger(smaller) improvement is obtained with less(more) labeled data.

**Table 2:** The description of experimental datasets used in the experiments on collaboration networks.

Datasets	Number of Instances	Number of Attributes	Number of Links	Number of Classes
DBLP-A	23,806	12,588	150,042	6
DBLP-B	16,020	8,595	95,108	6

## Conclusion

In this paper, we studied the label deficiency problem in collective classification (CC). We showed the protein function prediction problem from PPI networks can be formulated as a problem, and proposed an effective and novel Markov chain based CC learning algorithm, namely ICAM. It focuses on how to use labeled and unlabeled data to enhance the classification performance of PPI network data. Experimental results on two real-world Yeast PPI network datasets and two collaboration network datasets showed that our proposed ICAM method is effective in learning CC tasks in the paucity of labeled data. In future, we will consider other semi-supervised learning techniques for collective classification in PPI network data and we will also research on other complex biological networks, such as heterogeneous network classification.

## Competing interests

The authors declare that they have no competing interests.

## Authors' contributions

Q. Wu, M.K. Ng. and Y. Ye participated in designing the algorithm and drafted the manuscript. Q. Wu and R. Shi performed the implementations. S.S. Ho revised and finalized the paper. All authors read and approved the final manuscript.

## Declarations

The publication costs for this article were funded by the corresponding author (Y. Ye).

This article has been published as part of *BMC Bioinformatics *Volume 15 Supplement 2, 2014: Selected articles from the Twelfth Asia Pacific Bioinformatics Conference (APBC 2014): Bioinformatics. The full contents of the supplement are available online at http://www.biomedcentral.com/bmcbioinformatics/supplements/15/S2.

## References

[B1] Eisenberg D Marcotte EM Xenarios I Yeates TO Protein function in the post-genomic era Nature 2000 405 6788 823 826 10.1038/35015694 10866208

[B2] Pandey G Kumar V Steinbach M Meyers CL Computational Approaches to Protein Function Prediction 2012 Wiley-Interscience

[B3] Clare A King RD Predicting gene function in saccharomyces cerevisiae Bioinformatics 2003 19 suppl 2 42 49 14534170 10.1093/bioinformatics/btg1058

[B4] Borgwardt KM Ong CS Schönauer S Vishwanathan S Smola AJ Kriegel H-P Protein function prediction via graph kernels Bioinformatics 2005 21 suppl 1 47 56 10.1093/bioinformatics/bti1007 15961493

[B5] Sleator RD Walsh P An overview of in silico protein function prediction Archives of microbiology 2010 192 3 151 155 10.1007/s00203-010-0549-9 20127480

[B6] Altschul SF Evaluating the statistical significance of multiple distinct local alignments Theoretical and Computational Methods in Genome Research 1997 1 14

[B7] Sigrist CJ Cerutti L De Castro E Langendijk-Genevaux PS Bulliard V Bairoch A Hulo N Prosite, a protein domain database for functional characterization and annotation Nucleic acids research 2010 38 suppl 1 161 166 19858104 10.1093/nar/gkp885 PMC2808866

[B8] Finn RD Mistry J Schuster-Böckler B Griffiths-Jones S Hollich V Lassmann T Moxon S Marshall M Khanna A Durbin R Pfam: clans, web tools and services Nucleic acids research 2006 34 suppl 1 247 251 16381856 10.1093/nar/gkj149 PMC1347511

[B9] Sharan R Ulitsky I Shamir R Network-based prediction of protein function Molecular systems biology 2007 3 1 17353930 10.1038/msb4100129 PMC1847944

[B10] Chua HN Sung WK Wong L Exploiting indirect neighbours and topological weight to predict protein function from protein-protein interactions Bioinformatics 2006 22 13 1623 1630 10.1093/bioinformatics/btl145 16632496

[B11] Nabieva E Jim K Agarwal A Chazelle B Singh M Whole-proteome prediction of protein function via graph-theoretic analysis of interaction maps Bioinformatics 2005 21 suppl 1 302 310 10.1093/bioinformatics/bti1054 15961472

[B12] Brun C Chevenet F Martin D Wojcik J Gueénoche A Jacq B Functional classification of proteins for the prediction of cellular function from a protein-protein interaction network Genome biology 2004 5 1 6 6 14709178 10.1186/gb-2003-5-1-r6 PMC395738

[B13] Adamcsek B Palla G Farkas IJ Derényi I Vicsek T Cfinder: locating cliques and overlapping modules in biological networks Bioinformatics 2006 22 8 1021 1023 10.1093/bioinformatics/btl039 16473872

[B14] McDowell L Aha DW Semi-supervised collective classification via hybrid label regularization Proceedings of the 29th International Conference on Machine Learning 2012

[B15] Neville J Jensen D Iterative classification in relational data Proc AAAI-2000 Workshop on Learning Statistical Models from Relational Data 2000 13 20

[B16] Jensen D Neville J Gallagher B Why collective inference improves relational classification Proceedings of the Tenth ACM SIGKDD International Conference on Knowledge Discovery and Data Mining 2004 593 598

[B17] Macskassy SA Provost F Classification in networked data: A toolkit and a univariate case study The Journal of Machine Learning Research 2007 8 935 983

[B18] Shi X Li Y Yu P Collective prediction with latent graphs Proceedings of the 20th ACM International Conference on Information and Knowledge Management 2011 1127 1136

[B19] McDowell L Aha D Semi-supervised collective classification via hybrid label regularization 2012 975 982 arXiv preprint arXiv:1206.6467

[B20] Mcdowell LK Gupta KM Aha DW Case-based collective classification Proceedings of the 20th International FLAIRS Conference 2007 399 404

[B21] Macropol K Can T Singh A Rrw: repeated random walks on genome-scale protein networks for local cluster discovery BMC bioinformatics 2009 10 1 283 10.1186/1471-2105-10-283 19740439 PMC2748087

[B22] Li X Ng MK Ye Y Multicomm: Finding community structure in multi-dimensional networks IEEE Transactions on Knowledge and Data Engineering 2013 99 1

[B23] Wu Q Ng MK Ye Y Markov-miml: A markov chain-based multi-instance multi-label learning algorithm Knowledge and Information Systems

[B24] Ng MK Wu Q Ye Y Co-transfer learning via joint transition probability graph based method Proceedings of the 1st International Workshop on Cross Domain Knowledge Discovery in Web and Social Network Mining 2012 1 9 ACM

[B25] Tong H Faloutsos C Pan J-Y Random walk with restart: fast solutions and applications Knowledge and Information Systems 2008 14 3 327 346 10.1007/s10115-007-0094-2

[B26] Zelnik-manor L Perona P Self-tuning spectral clustering Advances in Neural Information Processing Systems 2004 1601 1608

[B27] Haveliwala TH Topic-sensitive pagerank: A context-sensitive ranking algorithm for web search IEEE Transactions on Knowledge and Data Engineering 2003 784 796

[B28] Cheng J Hatzis C Hayashi H Morishita S Page D Sese J Kdd cup 2001 report ACM SIGKDD Explorations Newsletter 2002 3 2 47 64 10.1145/507515.507523

[B29] Kong X Shi X Yu PS Multi-label collective classification SIAM International Conference on Data Mining (SDM) 2011 618 629

[B30] Schapire RE Singer Y Boostexter: A boosting-based system for text categorization Machine learning 2000 39 2-3 135 168

[B31] Zhou Z-H Zhang M-L Huang S-J Li Y-F Multi-instance multi-label learning Artificial Intelligence 2012 176 1 2291 2320 10.1016/j.artint.2011.10.002

[B32] Davis J Goadrich M The relationship between precision-recall and roc curves Proceedings of the 23rd International Conference on Machine Learning 2006 233 240

[B33] Chang C-C Lin C-J Libsvm: a library for support vector machines ACM Transactions on Intelligent Systems and Technology (TIST) 2011 2 3 27

